# Hidden in Plain Sight: A Rare 7q Deletion Masquerading as a Common Aneuploidy on Prenatal Ultrasound

**DOI:** 10.7759/cureus.98598

**Published:** 2025-12-06

**Authors:** Zainab Hananah Abang Abdullah, Mumtazah Mohd Alauddin, Suziela Mohamad, Aisha Fadhilah Abang Abdullah, Amilia Afzan Mohd Jamil

**Affiliations:** 1 Obstetrics and Gynaecology, Faculty of Medicine and Health Sciences, Universiti Putra Malaysia, Serdang, MYS; 2 Obstetrics and Gynaecology, Hospital Sultan Abdul Aziz Shah, Universiti Putra Malaysia, Serdang, MYS; 3 Paediatrics, Faculty of Medicine and Health Sciences, Universiti Putra Malaysia, Serdang, MYS

**Keywords:** 7q deletion, chromosomal microarray analysis, fetal growth restriction, prenatal diagnosis, ultrasound

## Abstract

Prenatal diagnosis of rare chromosomal microdeletions is challenging when fetal abnormalities mimic common aneuploidies. Traditional karyotyping may miss submicroscopic aberrations detectable by chromosomal microarray analysis (CMA). We report a case of 7q31.1-q32.3 microdeletion syndrome identified through CMA, highlighting its diagnostic value. A 28-year-old primigravida at 29 weeks presented with polyhydramnios, fetal growth restriction, short nasal bone, and cardiac abnormalities suggestive of trisomy. CMA revealed a 21.58-megabase deletion at 7q31.1-q32.3. She delivered at 37 weeks by cesarean section. The neonate developed respiratory distress requiring intensive care and was found to have an atrial septal defect, patent ductus arteriosus, pulmonary artery stenosis, ventriculomegaly, and feeding difficulties. This case demonstrates that prenatal ultrasound features suggestive of common aneuploidies may instead arise from other, less common genetic abnormalities. CMA offers superior diagnostic precision and should be considered in atypical prenatal presentations resembling aneuploidy.

## Introduction

Prenatal diagnosis has evolved significantly with the introduction of advanced cytogenetic techniques. Traditional karyotyping, while effective for detecting major chromosomal abnormalities and common aneuploidies such as trisomy 21, 18, and 13, has limitations in identifying submicroscopic chromosomal aberrations [1]. Chromosomal microarray analysis (CMA) has improved prenatal diagnostics by identifying clinically relevant microdeletions and microduplications at 50-100 kb resolution, far surpassing conventional karyotyping [2].

The clinical challenge arises when fetal ultrasound abnormalities suggest common aneuploidies, but standard screening tests yield normal results. In such cases, the underlying pathology may involve rare microdeletion or microduplication syndromes that present with overlapping phenotypic features. The 7q deletion syndrome, particularly involving the 7q31-q32 region, represents one such rare chromosomal disorder that can present with features resembling more common genetic conditions [3].

We present a case of prenatal diagnosis of 7q31.1-q32.3 microdeletion syndrome identified through CMA, highlighting the diagnostic superiority of this technique over conventional karyotyping and emphasizing its importance in contemporary prenatal care when common fetal structural abnormalities are detected.

## Case presentation

**Figure 1:**
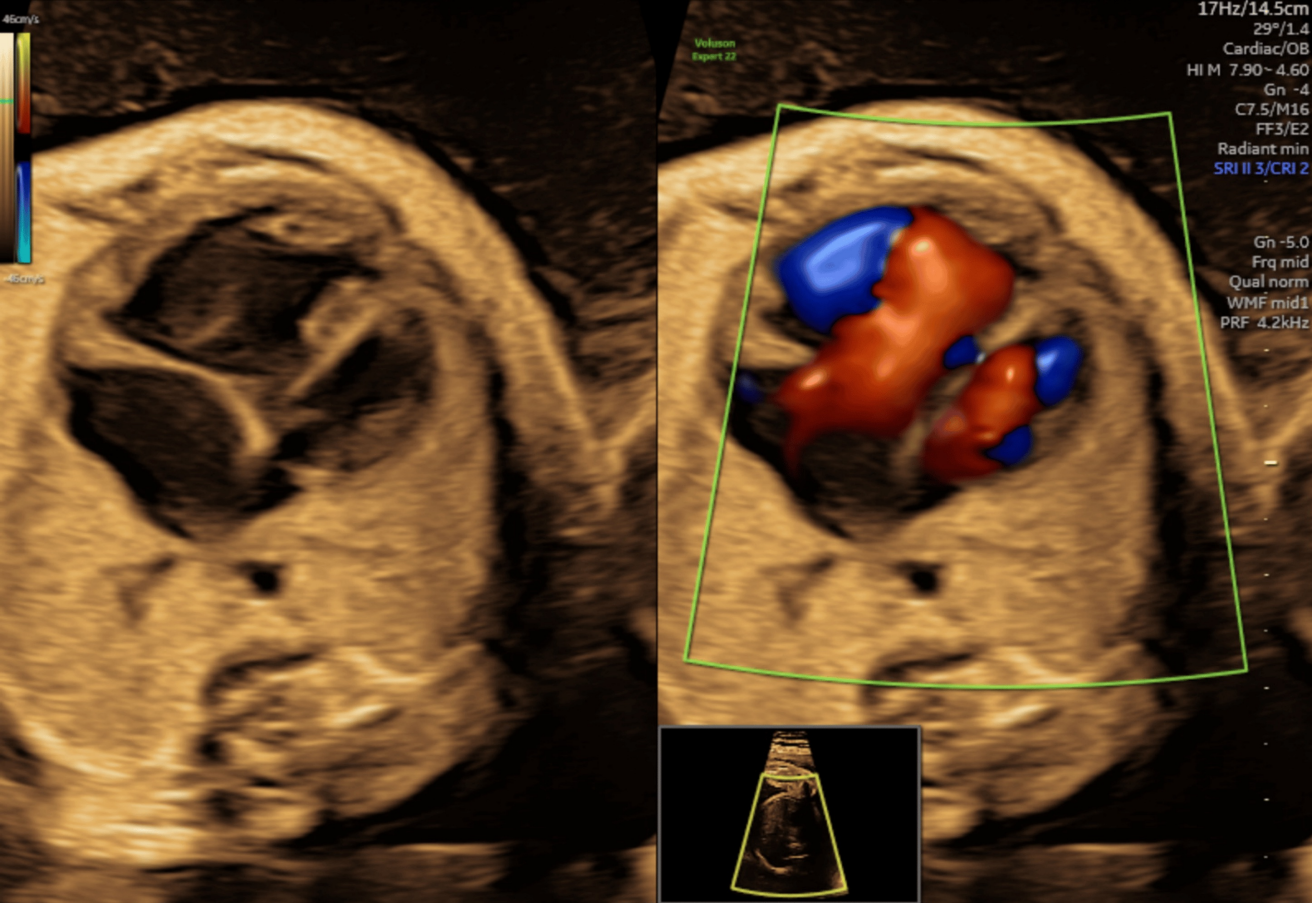
Fetal cardiac ultrasound image showing suspected congenital abnormality. Image taken by the author.

**Figure 2:**
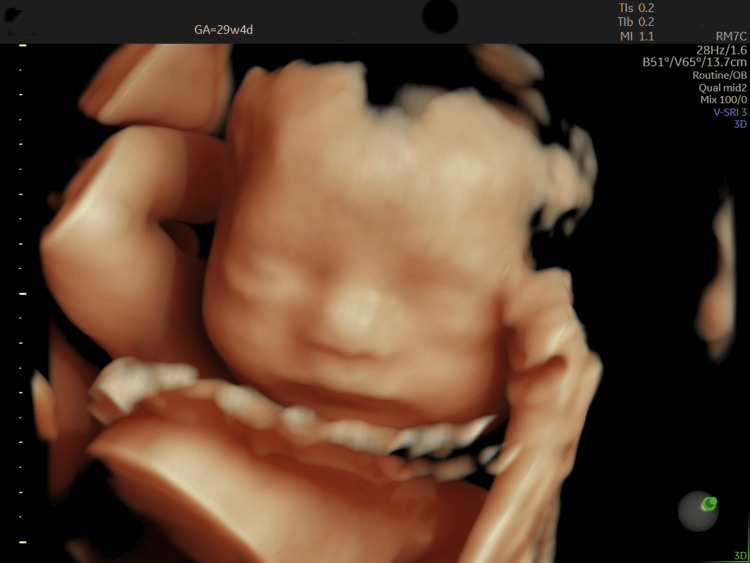
3D ultrasound depicting grossly normal craniofacial structure despite a 2D finding of a short nasal bone. Image taken by the author.

A 28-year-old primigravida was referred at 29 weeks of gestation after routine ultrasonography revealed multiple fetal anomalies. Her medical, antenatal, and family histories were unremarkable for genetic conditions. Ultrasound demonstrated polyhydramnios, fetal growth restriction (biometry <1st percentile for gestational age based on Hadlock nomograms [4]), a short nasal bone, and a suspected atrioventricular septal defect with left ventricular dilatation (Figure 1). Other structures were grossly normal (Figure 2).

**Figure 3:**
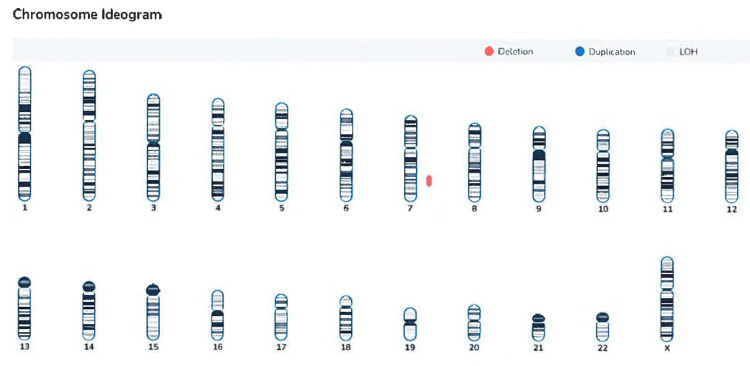
Chromosome ideogram demonstrating 7q31.1-q32.3 deletion. Figure reproduced with permission from the external laboratory.

Amniocentesis was performed and sent for CMA. The results revealed a significant finding: a 21.58 megabase (Mb) deletion spanning the 7q31.1-q32.3 region of chromosome 7 (Figure 3). This large interstitial deletion encompassed multiple genes known to be associated with developmental abnormalities and congenital anomalies.

Fetal echocardiography at 30 weeks of gestation revealed a large atrial septal defect (ASD) with right and left ventricular discrepancy, and suspicion of coarctation of the aorta. She underwent an elective lower segment cesarean section at 37 weeks of gestation due to early onset fetal growth restriction (FGR) with breech presentation and polyhydramnios, and delivered a female neonate, with a birthweight of 2.11 kg (<2nd percentile).

Initial newborn assessment revealed no obvious dysmorphic features on clinical examination. However, the neonate developed respiratory distress requiring non-invasive ventilatory support via continuous positive airway pressure. Postnatal echocardiography noted the presence of a large secundum ASD, small patent ductus arteriosus, right pulmonary artery stenosis, with pulmonary hypertension, and estimated pulmonary artery pressure of 25 mmHg. Repeated cranial ultrasonography was performed, which demonstrated ventriculomegaly, and is planned for brain MRI evaluation.

Currently at 1.5 months of age, the infant remains hospitalized and requires ongoing non-invasive ventilatory support due to decompensated heart failure with pulmonary hypertension secondary to a large ASD while awaiting transfer for definitive ASD closure. Growth parameters have shown improvement, with weight increasing from 2.11 kg at birth to 2.85 kg at current assessment; however, long-term direct feeding is an issue due to poor sucking. The parents are currently awaiting consultation with a clinical geneticist for comprehensive genetic counseling regarding the 7q31.1-q32.3 deletion syndrome, including discussion of long-term prognosis, developmental expectations, and recurrence risk for future pregnancies.

## Discussion

This case exemplifies the diagnostic challenges encountered in prenatal medicine when fetal abnormalities suggest common chromosomal disorders, but underlying rare microdeletion syndromes are the actual cause. 7q terminal deletion syndrome is a rare condition presenting with multiple congenital malformations, including abnormal brain and facial structures, developmental delay, intellectual disability, abnormal limbs, and sacral anomalies, but most literature focuses on postnatal diagnosis rather than prenatal detection [5]. CMA is cost-effective as a first-line test in fetuses with structural anomalies. Compared with traditional karyotyping, using CMA as a first-line approach increases diagnostic yield, detects clinically significant variants missed by karyotyping, and lowers the cost per genetic diagnosis despite higher per-test costs [6].

Table 1 shows a comprehensive review, adapted from a paper published in 2024 by Aneja et al. [7], which compiled ultrasound features associated with 7q deletions, including microcephaly, holoprosencephaly, and craniofacial abnormalities [7-12]. However, our case presented with a more limited phenotypic spectrum, primarily manifesting as fetal growth restriction and cardiac abnormalities, without the classical dysmorphic features typically associated with 7q deletion syndrome. The pattern of ultrasound findings in our patient, including polyhydramnios, short nasal bone, cardiac abnormalities, and a fetus with growth restriction, is classically associated with common aneuploidies such as trisomy 21, 18, or 13 [13].

**Table 1:** Reported cases of terminal 7q syndrome with antenatal ultrasound features. USG = ultrasonography; HPE = holoprosencephaly; IUGR = intrauterine growth retardation; TOF = tetralogy of Fallot; CDH = congenital diaphragmatic hernia; SUA = single umbilical artery. Source: Adapted from Aneja et al. (2024) [7] under the Creative Commons Attribution Non-Commercial - No Derivatives 4.0 International Deed.

Case	Gestational age at USG	Antenatal USG features	Cytogenetic location	Size of deletion
Aneja et al. (2024) [7]	12 weeks and 6 days	HPE, hypotelorism, cleft palate, absent nasal bone, SUA	7q36.2–36.3	5.5 Mb
Chen et al. (1999) [8]	23 weeks	HPE, proboscis, cyclopia, microcephaly, IUGR	7q36 and distal 3p	–
Chen et al. (2003) [9]	22 weeks	Microcephaly, TOF, IUGR	7q35	–
Chen et al. (2015) [10]	15 weeks	HPE, cyclopia, nuchal edema	7q36.1q36.3 and 3p26.3p22.1	43.68 Mb
Song et al. (2016) [11]	23 weeks	Hemivertebra with scoliosis in the thoracic spine, spinal stenosis	7q36.1q36.3	6.4 Mb
Zhao et al. (2022) [12]	21 weeks	HPE, cleft lip, cleft palate, CDH, subcutaneous cystic mass in neck	7q32.3qter	27.7 Mb

The 21.58Mb deletion spanning 7q31.1-q32.3 in our case represents a particularly large interstitial deletion encompassing numerous genes critical for neurodevelopment. 7q terminal deletion syndrome commonly includes developmental delay, growth restrictions, intellectual disability, and microcephaly, and is associated with holoprosencephaly but often presents as microforms [14]. The early-onset fetal growth restriction observed in our case may be attributed to the loss of genes within the deleted region that are essential for normal fetal growth and development [14]. The genes within the 7q31-q32 region are known to play crucial roles in neurological development and function [15].

While direct literature linking 7q deletions to swallowing dysfunction is limited, the neurological abnormalities associated with this syndrome, including developmental delays and brain malformations, can potentially affect fetal swallowing mechanisms, hence the persistent polyhydramnios and poor sucking reflex [16]. Similar to other chromosomal deletion syndromes, such as 22q11.2 deletion syndrome, where patients typically have trouble coordinating the suck/swallow/breath pattern, resulting in slow nipple feedings interrupted by gagging or regurgitation, the 7q deletion may have comparable effects on swallowing coordination and esophageal motility [16].

The neurological impact of 7q deletions on swallowing function represents an underexplored area that warrants further investigation, particularly given the potential implications for prenatal counseling and postnatal management planning. Continued research into rare genetic syndromes and their prenatal presentations will further enhance our ability to provide comprehensive genetic counseling and optimize perinatal care.

## Conclusions

This case demonstrates the diagnostic advantage of CMA over conventional karyotyping in detecting 7q31.1-q32.3 microdeletion syndrome, even when prenatal imaging suggests more common aneuploidies. CMA improves genotype-phenotype correlation and refines fetus-centered care planning, including optimized delivery, targeted post-delivery management, and family counseling.
